# Genome-Wide SNP Discovery from Transcriptome of Four Common Carp Strains

**DOI:** 10.1371/journal.pone.0048140

**Published:** 2012-10-26

**Authors:** Jian Xu, Peifeng Ji, Zixia Zhao, Yan Zhang, Jianxin Feng, Jian Wang, Jiongtang Li, Xiaofeng Zhang, Lan Zhao, Guangzan Liu, Peng Xu, Xiaowen Sun

**Affiliations:** 1 Centre for Applied Aquatic Genomics, Chinese Academy of Fishery Sciences, Beijing, People’s Republic of China; 2 Heilongjiang Fisheries Research Institute, Chinese Academy of Fishery Sciences, Harbin, People’s Republic of China; 3 Henan Academy of Fishery Sciences, Zhengzhou, Henan, People’s Republic of China; 4 National Fish Hatchery of Xingguo Red Carp, Xingguo, Jiangxi, People’s Republic of China; Auburn University, United States of America

## Abstract

**Background:**

Single nucleotide polymorphisms (SNPs) have been used as genetic marker for genome-wide association studies in many species. Gene-associated SNPs could offer sufficient coverage in trait related research and further more could themselves be causative SNPs for traits. Common carp (*Cyprinus carpio*) is one of the most important aquaculture species in the world accounting for nearly 14% of freshwater aquaculture production. There are various strains of common carp with different economic traits, however, the genetic mechanism underlying the different traits have not been elucidated yet. In this project, we identified a large number of gene-associated SNPs from four strains of common carp using next-generation sequencing.

**Results:**

Transcriptome sequencing of four strains of common carp (mirror carp, purse red carp, Xingguo red carp, Yellow River carp) was performed with Solexa HiSeq2000 platform. *De novo* assembled transcriptome was used as reference for alignments, and SNP calling was done through BWA and SAMtools. A total of 712,042 Intra-strain SNPs were discovered in four strains, of which 483,276 SNPs for mirror carp, 486,629 SNPs for purse red carp, 478,028 SNPs for Xingguo red carp and 488,281 SNPs for Yellow River carp were discovered, respectively. Besides, 53,893 inter-SNPs were identified. Strain-specific SNPs of four strains were 53,938, 53,866, 48,701, 40,131 in mirror carp, purse red carp, Xingguo red carp and Yellow River carp, respectively. GO and KEGG pathway analysis were done to reveal strain-specific genes affected by strain-specific non-synonymous SNPs. Validation of selected SNPs revealed that 48% percent of SNPs (12 of 25) were tested to be true SNPs.

**Conclusions:**

Transcriptome analysis of common carp using RNA-Seq is a cost-effective way of generating numerous reads for SNP discovery. After validation of identified SNPs, these data will provide a solid base for SNP array designing and genome-wide association studies.

## Introduction

Common carp (*Cyprinus carpio*) is a widespread freshwater fish of eutrophic waters in lakes and large rivers in Europe and Asia. The wild populations are considered vulnerable to extinction, but the species has also been domesticated and introduced into various environments worldwide. With cultural history of several thousand years, common carp becomes one of the most important food fish with over hundred strains and varieties in the world. Common carp and its closely related Cyprinidae species provide over 30% aquaculture production in the world [1]. Besides, common carp is also selected and kept for decorative purposes. There are abundant strains and local populations of common carp in China, including mirror carp, purse red carp, Xingguo red carp, Yellow River carp, Oujiang color carp, and many hybrid populations,. Due to the economical and ecological importance of common carp, genetic and genomic studies had been performed in the past decade, which focused on development of genetic markers [2]–[6] for breeding and genetic evaluation, construction of genetic maps [7], [8] and physical map [9], collection of a large set of ESTs [10]–[12] and microRNA [1], [13], construction of bacterial artificial chromosome (BAC) library [14] and collection BAC-end sequences (BES) [15], EST collection and transcriptome study [16], characterization of functional genes [17] and quantitative trait loci (QTL) analysis [18], [19], etc.

Recently, the genome of common carp had been sequenced and assembled with the next generation sequencing platforms [20], which marked the beginning of a new era on genetic selection and breeding of carps. Although a large set of microsatellite markers had been developed for linkage mapping, QTL analysis and association study, there are still no sufficient markers for whole genome association study. Single nucleotide polymorphism (SNP) markers could meet the needs on both marker numbers and genome coverage and serve as molecular “ruler” on the genome. With the development of genomic resources, abundant genome and transcriptome data had been collected and assembled in many model and economically important species. A huge number of SNPs had then been identified and developed from various species, for instance, cattle [21], [22], Arabidopsis [23],rice [24], maize [25], [26], chicken [27], pig [28], dog [29], enabled genome-wide association studies and genome selection of complex traits. In aquaculture species, however, large set of SNPs had been only developed only in a few species, including catfish [30], [31], Oyster [32], Altantic Salmon [33] and Atlantic Cod [34]. Only a limit number of SNPs are available for common carp, which had been used on linkage mapping and QTL analysis [35].

SNP identification relies on highly redundant sequence data of the specific genome regions. The next generation sequencing technologies build the base for large scale SNP identification. The genome-wide SNP screening and marker development were generally performed after whole genome had been sequenced. Alternatively, Reduced Representation Library (RRL) technology and high throughput transcriptome sequencing could also fulfill the purpose [36]. Comparing to the SNPs from RRL platform, SNPs identified from transcriptome are actually cDNA SNPs (cSNP) and directly associated with genes or functional regions in the genome. In the past decade, Expression Sequence Tags (ESTs) had been collected from many species for gene and genetic marker identification. cSNPs had been then identified from these ESTs as by-products for genetic analysis. However, low sequencing coverage limited cSNP discovery from ESTs until emerging of the next generation sequencing technologies. Recently, transcriptome analysis using the next generation sequencing technologies have been widely reported in many species, including several aquaculture species such as catfish [30], [37], [38], Atlantic cod [34], silver carp [39], pearl oyster [40], etc. RNA-Seq on Illumina platform could generate redundant transcriptome sequences with ultra-high read depth, guaranteeing large scale cSNP identification with high quality than ever.

Transcriptome sequencing and assembly of common carp had been completed and reported which could serve as reference for cSNP identification. In this study, RNA-Seq had been conducted in four distinct common carp strains. RNA-Seq data had been mapped onto reference transcriptome of common carp, and cSNP had been identified and characterized from these four strains. These cSNP are invaluable resource for genetic and genome research of carps, especially for the design and construction of high throughput SNP genotyping platform in the future.

## Results and Discussion

### Generation of Expressed Short Reads

Illumina sequencing was conducted to generate short sequence reads of expressed sequences. The cDNAs were sequenced on Illumina HiSeq2000 platform that generated 114.9 million paired-end reads for mirror carp, 111.4 million for purse red carp, 112.2 million for Xingguo red carp, 105.4 million for Yellow River carp with read length of 50 bp (Table 1).

**Table 1:** Summary of Illumina expressed short reads production and filtration.

	Mirror	Purse red	Xingguo red	Yellow River
Reads (×10^6^)	114.9	111.4	112.2	105.4
Bases sequenced (×10^9^)	5.74	5.57	5.61	5.27

### Alignment of Short Reads and SNP Identification

**Figure 1:**
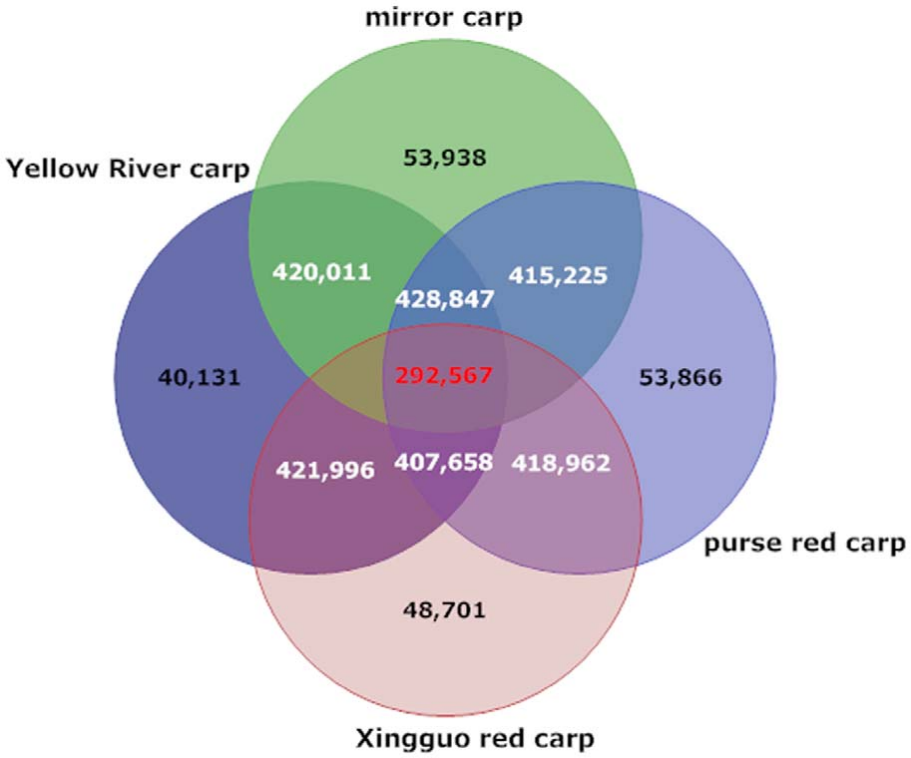
Venn diagram of non-redundant SNPs.

The short reads of RNA-Seq data from 4 strains had aligned onto reference transcriptome of common carp [41]. There are 50.8%, 47.5%, 47.4%, 48.2% of the short reads from mirror carp, purse red carp, Xingguo red carp and Yellow River carp mapped on reference transcriptome. The mapping ratio of mirror carp is slightly higher than that of the other three strains, as reference transcriptome were assembled based on mirror carp samples. Putative SNPs were then identified from 4 strains of common carp based on read depth and quality score of alignment results (see Methods). As summarized in Table 2, a total of 483,276 putative intra-strain SNPs were identified from mirror carp; 486,629 intra-strain putative SNPs were identified from purse red carp; 478,028 intra-strain putative SNPs were identified from Xingguo red carp; 488,281 intra-strain putative SNPs were identified from Yellow River carp. Almost two thirds of the putative SNPs were transitions in each strain, which is consistent with previous reports in other teleost fish [30], [33], [42]. Inter-strain putative SNPs were also identified which showed monomorphism in any strain of the comparison but showed polymorphism between two strains. There were 33,081 inter-strain putative SNPs between mirror carp and purse red carp, 30,846 between mirror carp and Xingguo red carp, 25,789 between mirror carp and Yellow River carp, 29,911 between purse red carp and Xingguo red carp, 29,536 between purse red carp and Yellow River carp, 27,427 between Xingguo red carp and Yellow River carp. Non-redundant SNPs were then identified from RNA-Seq data of all 4 strains. Those non-redundant SNPs in common carp were defined as [intra-strain(mirror carp∪purse red carp∪Xingguo red carp∪Yellow River carp)]+ [inter-strain(mirror carp vs. purse red carp)∪(mirror carp vs. Xingguo red carp)∪(mirror carp vs. Yellow River carp)∪(purse red carp vs. Xingguo red carp)∪(purse red carp vs. Yellow River carp)∪(Xingguo red carp vs. Yellow River carp)] in this study. A total of 712,042 putative non-redundant intra-strain SNPs and a total of 53,893 putative non-redundant inter-stain SNPs were identified from 4 strains of common carp. The Venn diagram (Figure 1) showed the shared intra-strain SNPs among all 4 strains. There were a total of 292,567 putative intra-strain SNPs shared among all 4 strains. Inter-strain putative SNPs were pooled and redundant SNPs were removed from the list. A total of 53,893 non-redundant inter-strain putative SNPs were also identified, which could be used for strain-specific marker development and relative applications. Thus, strain-specific SNPs were filtered, a total of 53,938 strain-specific SNPs were identified from mirror carp; 53,866 strain-specific SNPs were identified from purse red carp; 48,701 strain-specific SNPs were identified from Xingguo red carp; 40,131 strain-specific SNPs were identified from Yellow River carp.

**Table 2:** Statistics of intra-SNPs discovered from RNA-Seq data of four strains of common carp.

	SNP numbers	Transation	Transversion
Non-redundant intra-strain SNPs	712,042	470,892 (66.1%)	241,150 (33.9%)
Mirror carp	483,276		
Purse red carp	486,629		
Xingguo red carp	478,028		
Yellow River carp	488,281		

### SNP Classification

As reference transcriptome has been aligned to zebrafish EST database by ESTScan software [41], ORFs were identified and SNPs were classified to several categories including non-synonymous, synonymous, 5′-UTR and 3′-UTR. As shown in Table 3, among 712,042 putative non-redundant intra-strain SNPs, 285,612 SNPs were non-synonymous, 200,492 SNPs were synonymous, 35,812 SNPs in 5′-UTR, 51,238 SNPs in 3′-UTR, while 138,888 SNPs were not defined. Non-synonymous SNPs were further classified to several categories, missense, pre-terminated, and skip-stop-codon, with numbers of 261,267, 14,681, 9,667, respectively. Meanwhile, 53,893 inter-SNPs were also classified as above. 18,875 SNPs were non-synonymous, 16,349 SNPs were synonymous, 2,291 SNPs in 5′-UTR, 5,705 SNPs in 3′-UTR, while 10,673 SNPs were not defined.

**Table 3:** Classification of intra-strain SNPs.

SNP classification	Number of intra-strain SNPs
5′ UTR	35,812
3′ UTR	51,238
Coding region	486,104
synonymous	200,492
non-synonymous	285,612
pre-terminated	14,681
skip-stop-codon	9,664
mis-sense	261,267
Undefined	138,888
Total	712,042

### Minor Allele Frequency Distribution

Minor allele frequency (MAF) is an importance factor for SNP loci evaluation.

**Figure 2:**
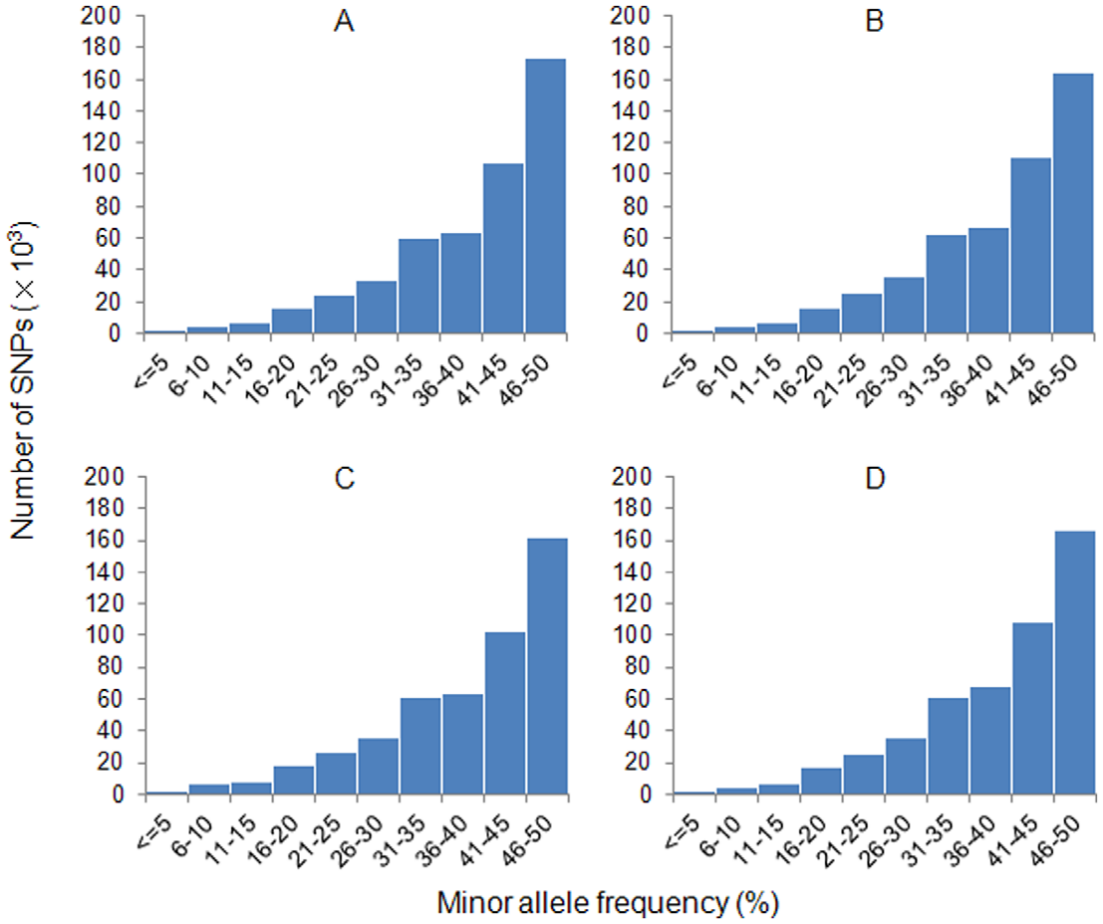
Distribution of minor allele frequencies of SNPs identified for four strains of common carp. A: strain-specific SNPs in mirror carp; B: strain-specific SNPs in purse redcarp; C: strain-specific SNPs in Xingguo red carp; D: strain-specific SNPs in Yellow River carp. The X-axis represents the SNP sequence derived minor allele frequency in percentage, while the Y-axis represents the number of SNPs with given minor allele frequency.

For each strain, MAF of SNPs were calculated and distribution was plotted (Figure 2).

As MAF results were calculated from transcriptome data, they may reflect relative abundance of transcription product of two alleles. However, they are probably not linear with real genome allele frequencies as many factors are involved in transcription progresses.

### SNP Distribution among Contigs and Genes

**Figure 3:**
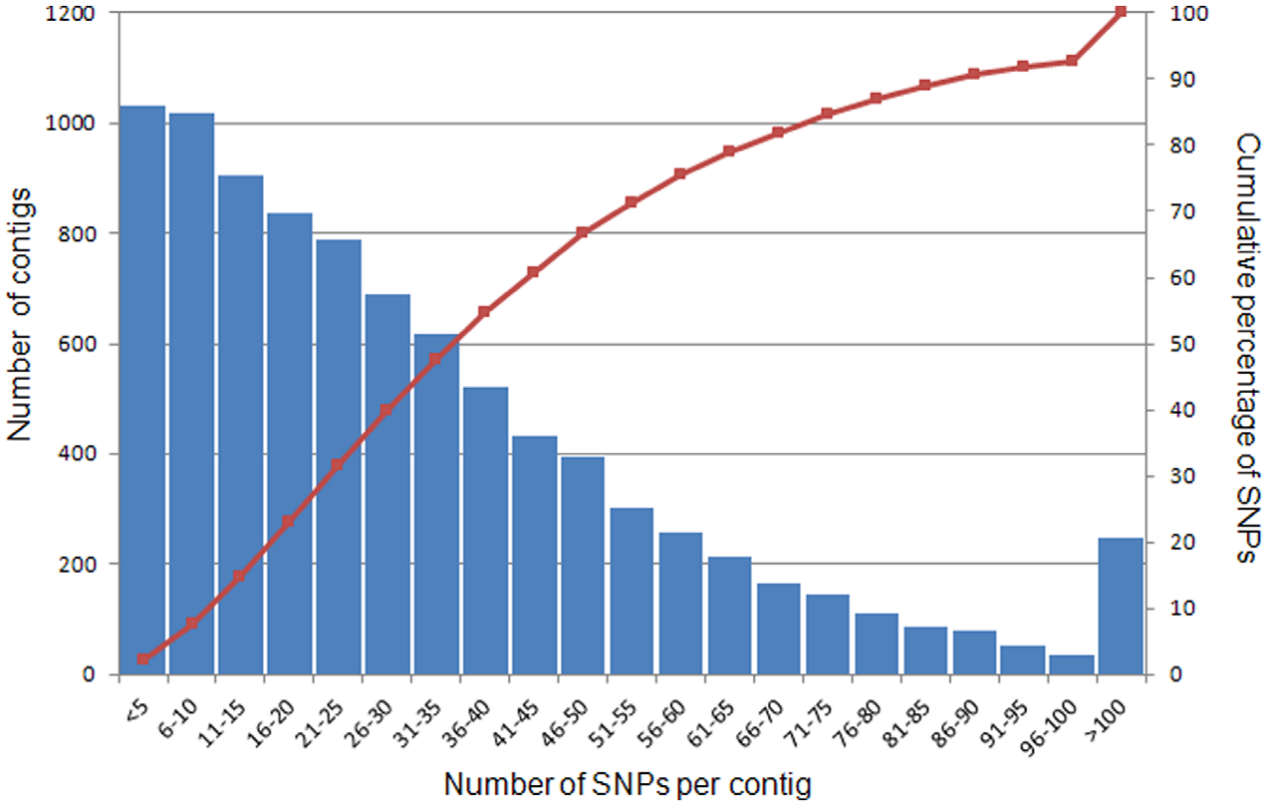
SNP distribution among contigs. The X-axis represents contig size (number of SNPs per contig). The curved line denotes the cumulative percentage of SNPs assembled.

SNPs distribution is important for consideration of coverage using SNP markers. Here we analyzed SNPs distribution among all contigs of reference transcriptome, which was shown in Figure 3. The majority of contigs have fewer than 50 SNPs per contig, consisting of over 65% of total SNPs.

**Figure 4:**
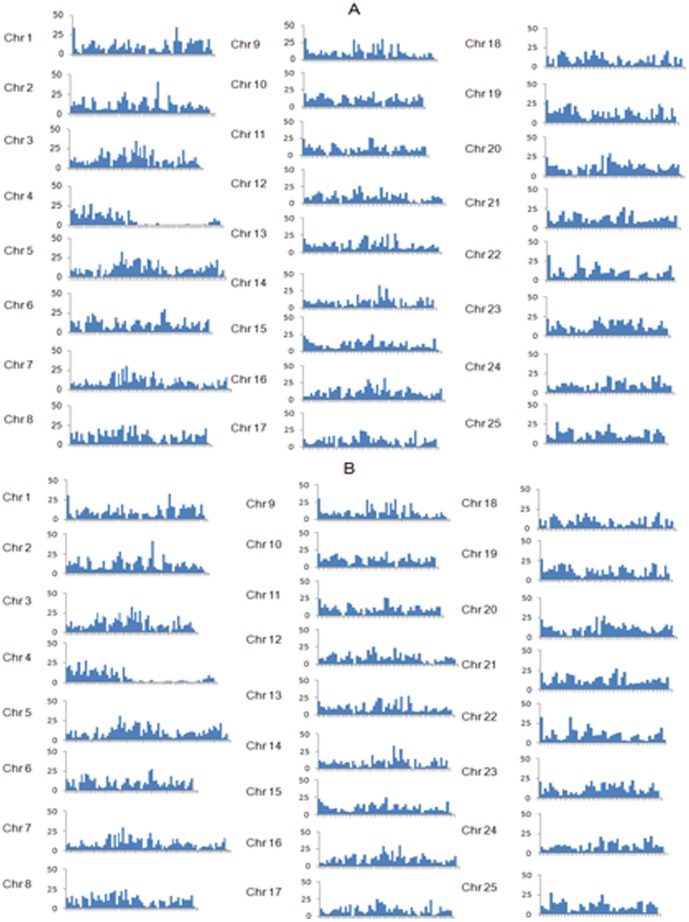
Comparative analysis of the all genes and genes containing SNPs on 25 chromosomes of the zebrafish genome. Each of the 25 zebrafish chromosomes was laid out in the X-axis with one million base pairs intervals, and the number of all genes (A) and genes contained with filtered SNPs (B) residing in the interval was plotted on the Y-axis.

Comparative analysis was applied using zebrafish genome as reference (Figure 4), showing distribution of 14,621 total genes and 13,706 genes containing SNPs on 25 chromosomes of zebrafish genome. Each of the 25 zebrafish chromosomes was laid out in the X-axis with one million base pairs intervals, and the number of genes contained with filtered SNPs residing in the interval was plotted on the Y-axis. More than 900 genes were screened with no SNPs according to our results, indicating that these genes may be very conservative and potentially house-keeping genes.

### Gene Ontology and KEGG Pathway Analysis

**Figure 5:**
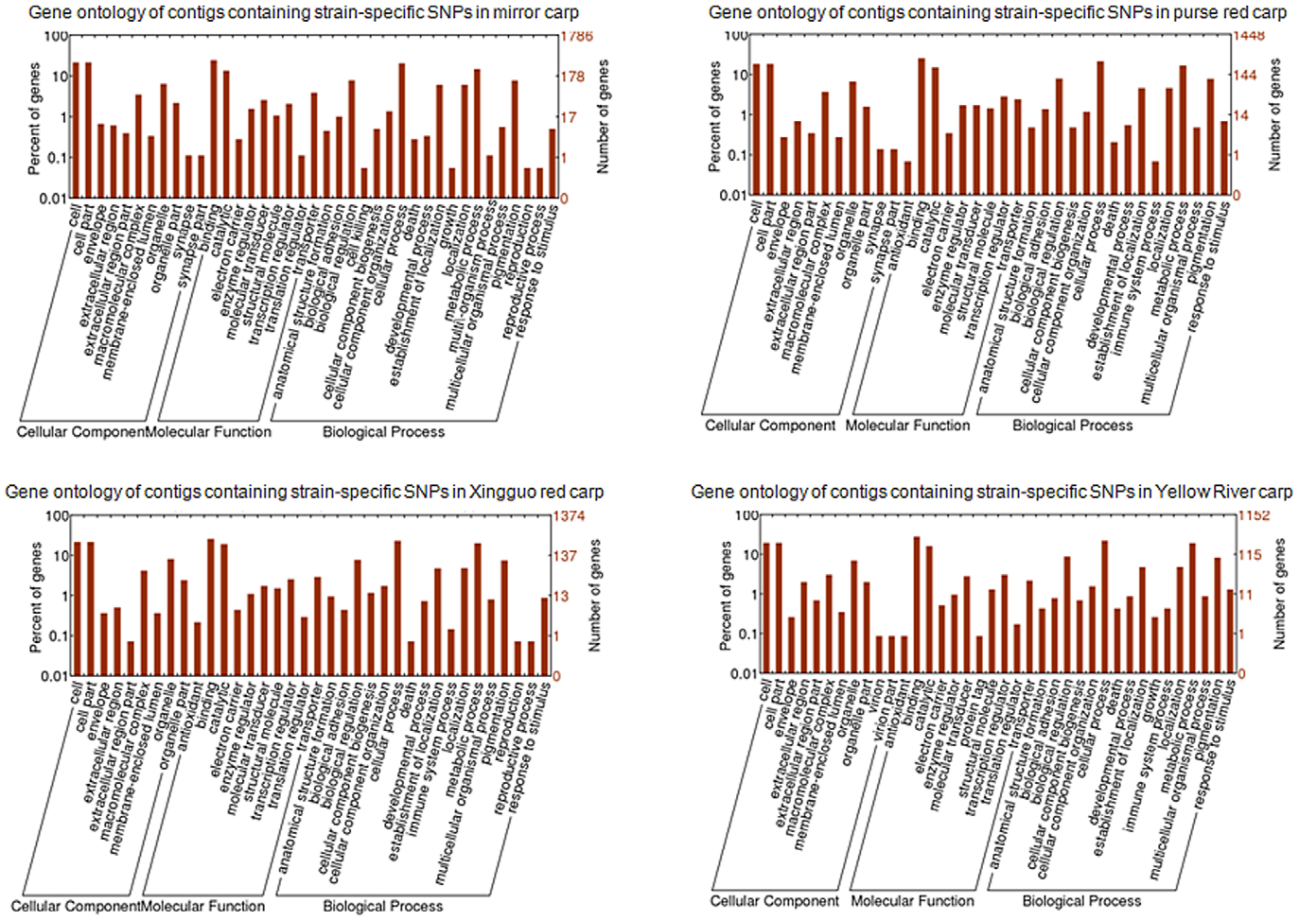
Gene ontology of contigs containing strain-specific SNPs.

As gene ontology (GO) analysis has been conducted on assembled transcriptome sequences by using InterProScan (http://www.ebi.ac.uk/Tools/pfa/iprscan/) and integrated protein databases [41], contigs containing non-synonymous SNPs were then extracted and annotated by previous annotation results. Annotated contigs in WEGO native format were imported into BGI WEGO program and GO annotations were plotted (http://wego.genomics.org.cn). As shown in Figure 5, the GO terms associated with contigs containing strain-specific non-synonymous SNPs in each strain were obtained for describing biological processes, molecular functions and cellular components.

As KEGG analysis of reference transcriptome has been conducted by colleagues [41], KEGG results of contigs containing strain-specific non-synonymous SNPs were extracted for functional categorization and annotation. Enzyme commission (EC) numbers were assigned to 183 unique genes for mirror carp, 200 unique genes for purse red carp, 172 unique genes for Xingguo red carp, 138 unique genes for Yellow River carp, which were categorized into different functional groups (Table 4). Comparison among KEGG results of strain specific contigs containing strain-specific non-synonymous SNPs could serve as important and valuable resources for gene identification and functional analysis of strain specific traits in common carp genetics and genomics.

**Table 4:** KEGG of genes with strain-specific SNPs in 4 strains of common carp.

KEGG categories represented	Number of genes			
	Mirror	Purse red	Xingguo red	Yellow River
Cellular Processing				
Communication	18	17	12	10
Growth death	13	15	12	5
Motility	10	11	8	4
Transport catabolism	21	25	19	15
Environmental Processing				
Membrane transport	1	2	0	0
Signal transduction	32	27	33	11
Signaling molecules interaction	11	7	8	10
Genetics				
Folding sorting degradation	16	22	28	13
Replication repair	9	4	7	6
Transcription	14	11	9	10
Translation	25	25	28	14
Metabolism				
Amino acid	5	5	6	5
Biosynthesis secondary	1	0	0	2
Carbohydrate	37	44	37	38
Cofactors vatamins	4	9	6	4
Energy	12	10	7	9
Glycan biosynthesis	7	5	8	3
Lipid	7	8	15	16
Nucleotide	8	8	6	8
Other amino acid	2	7	3	5
Terpenoids polyketides	1	0	1	0
Xenobiotics biodegradation	3	3	2	3
Organismal System				
Circulatory	8	8	6	3
Development	12	10	8	12
Digestive	13	8	7	12
Endocrine	12	13	16	12
Environmental adaption	0	3	2	0
Excretory	8	6	6	1
Immune	17	32	23	18
Nervous	14	15	16	12
Sensory	3	3	2	0

### SNP Validation

As SNPs were derived from bioinformatics analysis of transcriptome data, experimental results were needed for validation of our results. In this study, a total of 25 non-synonymous SNPs were randomly selected for validation. For each strain, DNA of 10 fish for was pooled as 1 sample. Totally 4 DNA pools were used for experiments. Among all 25 SNPs, 12 SNPs were proved and 13 SNPs were not found. Related genes containing true SNPs were shown in Table 5. This result showed that our SNPs from transcriptome analysis were convincible.

**Table 5:** SNPs validated by experiment.

Genes containing validated SNPs	Contig position	Ref allele	SNP allele
SKI3; superkiller protein 3	951	C	A
CDK5; cyclin-dependent kinase 5	136	G	T
TLR1; toll-like receptor 1	329	T	C
ADRBK; beta-adrenergic-receptor kinase	1029	C	T
glutathione S-transferase	130	T	C
MAP3K2; mitogen-activated protein kinase kinase kinase 2	1762	C	T
CYP51; cytochrome P450, family 51 (sterol 14-demethylase)	1126	T	C
TRIP10; thyroid hormone receptor interactor 10	918	G	A
ADCY3; adenylate cyclase 3	146	C	T
CSNK2A; casein kinase II subunit alpha	1153	C	T
HTR1; 5-hydroxytryptamine receptor 1	278	T	C
CES2; carboxylesterase 2	641	A	G

## Methods

### Ethics Statement

This study was approved by the Animal Care and Use committee of Centre for Applied Aquatic Genomics at Chinese Academy of Fishery Sciences.

### Sample Collection and RNA Isolation

Four common carp stains were sampled from distinct breeding stocks or population, including mirror carp from Heilongjiang Fishery Research Institute, Yellow River carp from Henan Fishery Research Institute, Xingguo red carp from National Fish Hatchery of Xingguo Red Carp at Xingguo, and purse red carp from National Fish Hatchery of Purse Red Carp at Wuyuan. Tissue samples of brain, skin, gill, blood, head kidney and muscle were collected from 18 individuals of each strain and immediately placed in 2 ml RNAlater (Qiagen, Hilden, Germeny) and kept at −20°C until RNA extraction. Total RNA was isolated from 24 samples using TRIZOL (Invitrogen, Carlsbad, CA, USA) with DNase I following manufacturer’s protocol. Integrity and size distribution of all samples were checked with Bioanalyzer 2100 (Agilent technologies, Santa Clara, CA, USA).

### cDNA Library Construction Illumina Sequencing

Sequencing of 24 samples was conducted in HudsonAlpha Genomic Services Laboratory (Huntsville, AL, USA). Briefly, 100 ng of total RNA was used for cDNA synthesis using Ovation RNA-Seq (NuGEN Technologies, SanCarlos, CA). The cDNA was then used for Illumina library construction. Adaptors were ligated to the cDNA which had been end-repaired. Each prepared tissue cDNA was sequenced with 50-bp paired-end reads on HiSeq2000. The following analysis of image data and signaling data were processed using the Illumina Pipeline Software according to the manufacturer’s instructions.

### SNP Identification

The raw reads were exported in FASTQ format, which were used as imported files for SNP calling. BWA and SAMtools software were applied to align reads to transcriptome reference and call SNPs. Filtering threshold was set as bellowing, read depth no less than 10, quality score no less than 20. The default parameter was used for quality control of flanking sequences in the step of “mpileup”.

### SNP Validation

Flanking sequences of selected SNPs were extracted and PCR primers were designed. Four samples were used as templates for PCR validation, and each sample was a DNA mixture of ten fish of a strain. PCR products were then sequenced by Sanger method and sequencing results were analyzed by DNASIS MAX v1.0 (Hitachi Solutions America, South San Francisco, CA, USA).

### Conclusions

In this study, the transcriptome of four strains of common carp were sequenced with Illumina HiSeq2000 platform, and large numbers of SNPs were discovered with an assembled reference transcriptome. Overall, these SNPs identified in this study provide useful resources for subsequent SNP array designing, genome-wide association studies and relative genetic research.
